# Low-Velocity Impact Behavior of Interlayer/Intralayer Hybrid Composites Based on Carbon and Glass Non-Crimp Fabric

**DOI:** 10.3390/ma11122472

**Published:** 2018-12-05

**Authors:** Chen Zhang, Yunfei Rao, Zhe Li, Wei Li

**Affiliations:** 1 College of Textiles, Donghua University, NO.2999, Northern Renmin Road, Shanghai 201620, China; 13381791726@163.com (C.Z.); ryf@mail.dhu.edu.cn (Y.R.); lizheaptx@gmail.com (Z.L.); 2 Key Lab of Textile Science & Technology, Ministry of Education, NO.2999, Northern Renmin Road, Shanghai 201620, China; 3 Center for Civil Aviation Composites, NO.2999, Northern Renmin Road, Shanghai 201620, China

**Keywords:** carbon/glass hybrid composites, interlayer hybrid, intralayer hybrid, low-velocity impact behavior, impact damage

## Abstract

Composites have gained wide use in structural applications; however, they are sensitive to impact damage. The use of hybrid composites is an effective way to overcome this deficiency. The effects of various hybrid structures of interlayer and intralayer warp-knitted fabrics with carbon and glass fibers on the low-velocity impact behavior of composite laminates were studied. Drop-weight impact tests were conducted on two types of interlayer, sandwich and intralayer hybrid composite laminates, which were compared with homogenous composite laminates. During low-velocity impact tests, the time histories of impact forces and absorbed energy by laminate were recorded. The failure modes were analyzed using the micro-CT (computed tomography) and C-scan techniques. The results revealed that the hybrid structure played an important role in peak force and the absorbed energy, and that the hybrid interface had an influence on damage modes, whereas the intralayer hybrid composite laminate damage was affected by the impact location. The intralayer hybrid laminate with C:G = 1:1 exhibited better impact resistance compared to the other hybrid structures.

## 1. Introduction

Composites are widely used in the fields of aviation and spaceflight because of their excellent specific strength and modulus. However, they are very sensitive to low-velocity impact damage, causing a potential threat as barely visible impact damage (BVID) [1,2,3], which is defined as impacts that show minimal surface damage but which can cause the internal structure to suffer a complex failure [4]. In addition, BVID can reduce the residual strength of the structure significantly [5]. Hybrid composites are made of two or more different types of fibers in a common matrix. The hybrid structure mainly includes the interlayer and intralayer hybrids, and the sandwich structure is a special form of the interlayer hybrids [6,7]. Intralayer hybrids are obtained by co-weaving and blending with multiple fibers in each layer [8,9]. The purpose of hybridization is to obtain a new material retaining the advantages of its constituents. Glass fiber has low fracture strength and modulus, but carbon fiber has high stiffness and strength [10,11]. Thus, hybrids including carbon fibers are fabricated most frequently with glass fibers, which are typically reported to improve most mechanical properties [12,13]. This is especially true for the next generation of commercial aircraft, wind-power blades, and some lightweight car parts.

Various studies on the low-velocity impact behavior of hybrid composites have been conducted, concerning the sample size, material, and impactor type, most of which having focused on the effect of the stacking sequence [14], especially for the interlayer hybrid structure [15]. Jiang et al. [16] compared the energy absorption in the sinusoidal plate with different stacking sequences, where it was shown that different stacking sequences had significant influences on failure modes and energy absorption capability. Riccio et al. [17] analyzed the mechanical behavior of laminates with different impact energy, and it was discovered that matrix damage to the single ply was obviously influenced by the given stacking sequence. In the work carried out by Fiore et al. [18], the aging resistance of jute-basalt interply hybrid laminates was studied, and it was found that the sandwich structure of the basalt hybrid performed best in terms of their mechanical performance compared to other generic composites. González et al. [19] evaluated the drop-weight impact response of interply hybrid laminates manufactured using polymer matrix composite materials. In a study by Özben [20], the effect of the misalignment angle of adjacent layers was examined by using two different fibers at the impact surface. The results showed that the impact performance of the laminate with glass fiber in the outer layer was greatly affected by the layer angle, whereas the carbon fiber at the outer layer could decrease the deformation of the laminate by increasing the misalignment angle. Sarasini et al. [21] compared the impact and bending response of basalt/carbon hybrid composites and found that the bending performance of the sandwich hybrid structure was good, whereas the damage tolerance of the interlayer hybrid structure was better. Swolfs et al. [22] proposed that an improvement in impact performance could be achieved by increasing the fracture toughness, and the impact resistance of hybrid composites was superior to the predicted value via hybrid law.

Extensive studies have been carried out to investigate the impact damage of hybrid composites [23,24]. Zhu et al. [25] assessed the impact behavior of Ti/M40 hybrid composites and found that the poor interlaminar forces were the main reason for the damage failure. Sayer et al. [26] used three materials including carbon fiber, glass fiber, and aramid fiber to design the hybrid composites and found that the delamination and fiber breakage on the bottom surface were the major damage mechanism. Sun et al. [27] proposed five types of aramid/polyethylene hybrid structures and found that the impact damage area was closely related to the hybrid structure, and that the damage area of the interlayer hybrid structure with aramid fiber on the impact surface was relatively small. Yang et al. [28] reported that the anti-delamination performance of carbon/glass interlayer hybrid laminates was correlated with the deformation properties of two fibers.

Most of the previous studies emphasized the interlayer hybrid structures. However, the low-velocity impact properties on intralayer hybrid composites have been rarely investigated due to the complex processing [29,30]. The fibers in non-crimp fabrics (NCFs) without buckling are all linked by knitted yarns, and the impact resistance can be improved by using the appropriate hybrid structure [31].

In this study, the low-velocity impact properties of carbon/glass fiber interlayer, sandwich and intralayer hybrid composite laminates were investigated. Load and energy versus time curves were recorded, and the important impact parameters like absorbed energy and peak load were studied in detail. In addition, the impact damage mechanisms of different hybrid structures were revealed by visual inspection, C-scan, and micro-CT techniques.

## 2. Materials and Methods

### 2.1. Materials

**Figure 1:**
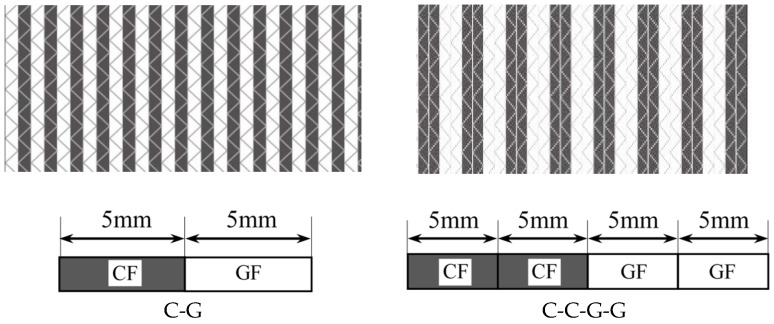
Schematic structure of two interlayer hybrid fabrics (C:G = 1:1).

Four types of hybrid NCFs, including carbon fiber fabric, glass fiber fabric, and two kinds of intralayer hybrid fabrics (with the same C-G ratio) were prepared, as is shown in Table 1. The carbon fiber was supplied by TORAY Inc. (Tokyo, Japan), the glass fiber was provided by CPIC glass fiber Inc., (Chongqing, China), and the epoxy resin was from SWANCOR Inc. (Shanghai, China). Two intralayer hybrid fabrics were supplied by CEDAR Composites Technology Inc. (Shanghai, China) and are shown in Figure 1.

### 2.2. Laminates

The laminate was placed by hand on 100 mm × 150 mm plates with the quasi-isotropic stacking sequences [0/90]_2S_ as recommended in ASTM-7167. The interlayer hybrid structures were designed according to the various stacking sequences of carbon fiber and glass fiber layers, and the intralayer hybrid structures consist of C-G and C-C-G-G fabrics as shown in Table 2. The vacuum-assisted resin infusion (VARI) process was adopted to prepare composite specimens, and the curing condition was 60 °C for 7 h as the resin would react completely.

### 2.3. Experiments

**Figure 2:**
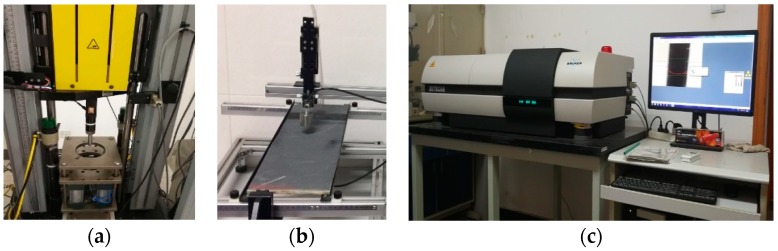
(**a**) INSTRON Dynatup 9250HV, (**b**) C-scan instrument, (**c**) Micro-CT scan system.

For low-velocity impact testing, the machine INSTRON Dynatup 9250HV (INSTRON Inc., Shanghai, China) employed with a hemispherical tup with a diameter of 12.7 mm (Figure 2a), the sample size was maintained at 100 mm × 150 mm with a thickness of 6 mm, and five samples of each laminate were tested at 30 J and 50 J according to ASTM D-7136. The data provided by the INSTRON Dynatup 9250HV were the evolution of the impact events, including the history of the tup displacement and velocity, as well as the energy and load history of the specimens [32]. Internal damage of specimens was studied using the C-scan method (Figure 2b, Japan Probe Co., Ltd, Kanagawa, Japan), and the cross-section damage visualization was performed by a Bruker SkyScan1172-Micro-CT (Figure 2c, Bruker Inc., Brussels, Belgium). The sample was cut down to 15 mm × 15 mm to improve CT-scan quality; meanwhile, according to the thickness of the samples, the camera pixel size was 1 mm and the source voltage was 100 kV.

## 3. Results and Discussion

### 3.1. Low-Velocity Impact Response

**Figure 3:**
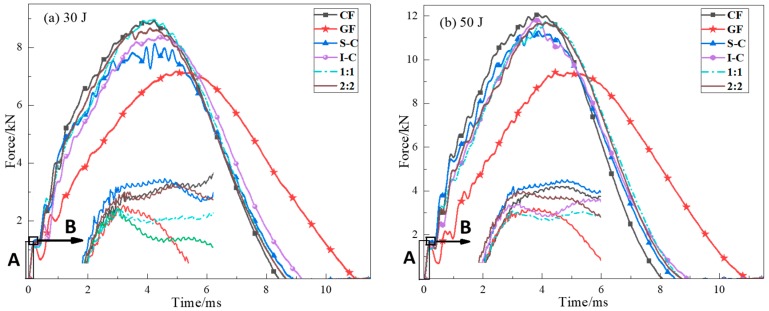
Force vs. time response of composite specimens at (**a**) 30 J and (**b**) 50 J.

**Figure 4:**
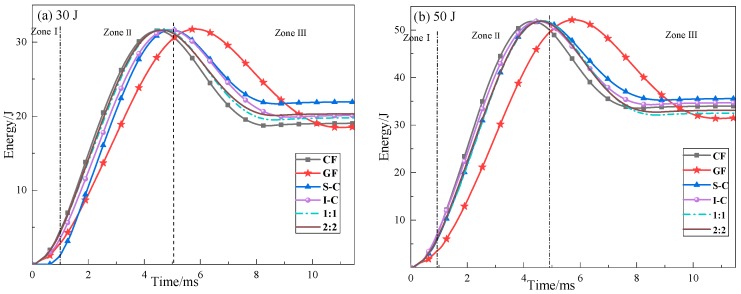
Energy vs. time response of composite specimens at (**a**) 30 J and (**b**) 50 J.

Figure 3 and Figure 4 show the load and energy as a function of the contact time responses for all kinds of specimens at 35 J and 50 J impact energy, respectively. Time versus force (T-F) curves provide damage initiation and growth regarding the low-velocity impact behavior of different composite laminates. It was revealed that each curve exhibited a similar increasing linearly phase in region A, and a sudden drop at point B was observed right after the region A. Point B is called the Hertzian failure point [33], which denotes the starting point of damage mainly in the form of matrix cracking and interlaminar delamination. The point B of GF and 1:1 was low, whereas that of CF, S-C, and 2:2 was higher, and the point B of the S-C was the highest. The curve of laminate with carbon fiber as the impact surface had a higher slope and fluctuation. In contrast, the curves of GF and the intralayer hybrid structure were smoother, and the curve fluctuation of 1:1 was higher than that of 2:2, the smoother curves indicating less damage during the impact event. Fmax (peak load) represents the maximum load that a composite laminate can bear before experiencing major damages during the contact. The S-C exhibited a ”plateau” near the peak force, whereas I-C showed a “cliff-drop” at the 50 J impact energy. It is expected that I-C suffered critical structure damage compared to S-C.

Impact test results showed that no penetration of the impactor to the specimens was observed. Figure 4 shows the evolution of the absorbed energy for each laminate under 30 J and 50 J impact energy (T-E). The absorbed energy is the energy transferred into the specimen as permanent damage, which depends on the type and size of the damage area. The curves can be divided into three distinct zones [34]. In the first stage (Zone I), the values of absorbed energy were slightly low. This may be attributed to the small indentation and deformation along the thickness direction after impact. In the second stage (Zone II), the T-E curves exhibited an increased slope, indicating greater internal damage including an increase in the contact area between the impactor and the specimen and the delamination area. In the third stage (Zone III), the elastic energy released by the laminate helped the impactor to rebound. The peak point refers to the impactor velocity as being zero, and the final constant value corresponds to the energy absorbed by the sample.

**Figure 5:**
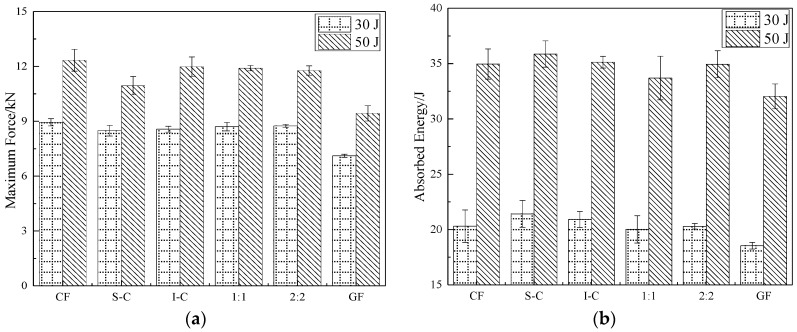
Impact parameters of all kinds of composite laminates: (**a**) peak load, (**b**) absorbed energy.

The key parameters including peak load and absorbed energy obtained from different hybrid structures are summarized in Figure 5. The peak load of I-C was higher than the other hybrid laminates, which was due to the carbon fiber modulus being higher than that of glass fiber, and the elongation fracture of glass fiber was two times higher than that of carbon fiber. Carbon fiber could resist the impactor drop used as skins; meanwhile, the next glass fiber would absorb energy through its deformation during the impact event. The intralayer hybrid structure had both in-plane and interlayer hybrid interfaces, leading to a stronger hybrid synergistic effect, and the peak load was nearly the same as that of I-C. In addition, the peak load of the 1:1 was larger than that of the 2:2, due perhaps to the small spacing of in-plane hybrid interfaces (5 mm < 10 mm) which can effectively prevent the impactor fall, thereby improving the impact resistance.

The effect of the hybrid structure on the energy absorption was significant. The S-C showed the maximal energy absorption (9% higher than CF and 17% higher compared with GF). Glass fiber has excellent toughness properties, leading to a protective effect on the inner carbon fiber, and the damage may be mainly attributed to the fiber-matrix debonding and the bottom ply delamination, causing less absorbed energy than the fiber breakage of S-C. The energy absorption of intralayer hybrid laminates depends on the internal damage which is related to the number of C-G hybrid interfaces per unit area. The C-G hybrid interfaces in 2:2 laminate per unit area were fewer, leading to a higher energy absorption.

### 3.2. Damage Analysis

**Figure 6:**
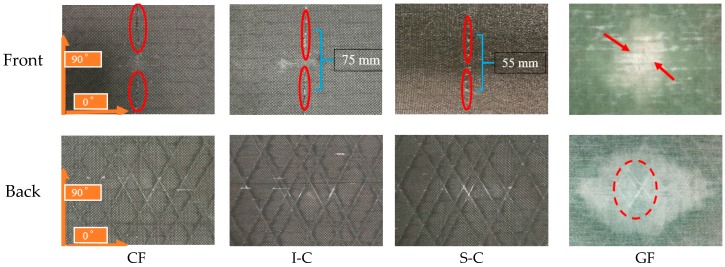
Front and back view photographs of composite specimens after impact at 50 J.

The front and back surfaces of the interlayer hybrid specimens under 50 J impact are shown in Figure 6. By visual inspection, all configurations exhibited a contact-induced indentation with fiber breakage around the impact point. The carbon fiber in the CF laminate exhibited an obvious transverse tearing (marked in red in the figure), as well as the I-C and S-C, and the longer transverse crack is to be noticed on the top ply of I-C, indicating a critical structure damage. The glass fiber in the GF laminate showed a smaller transverse shear damage but exhibited an obvious matrix crack. The visual inspection results were similar to the 30 J impact.

**Figure 7:**
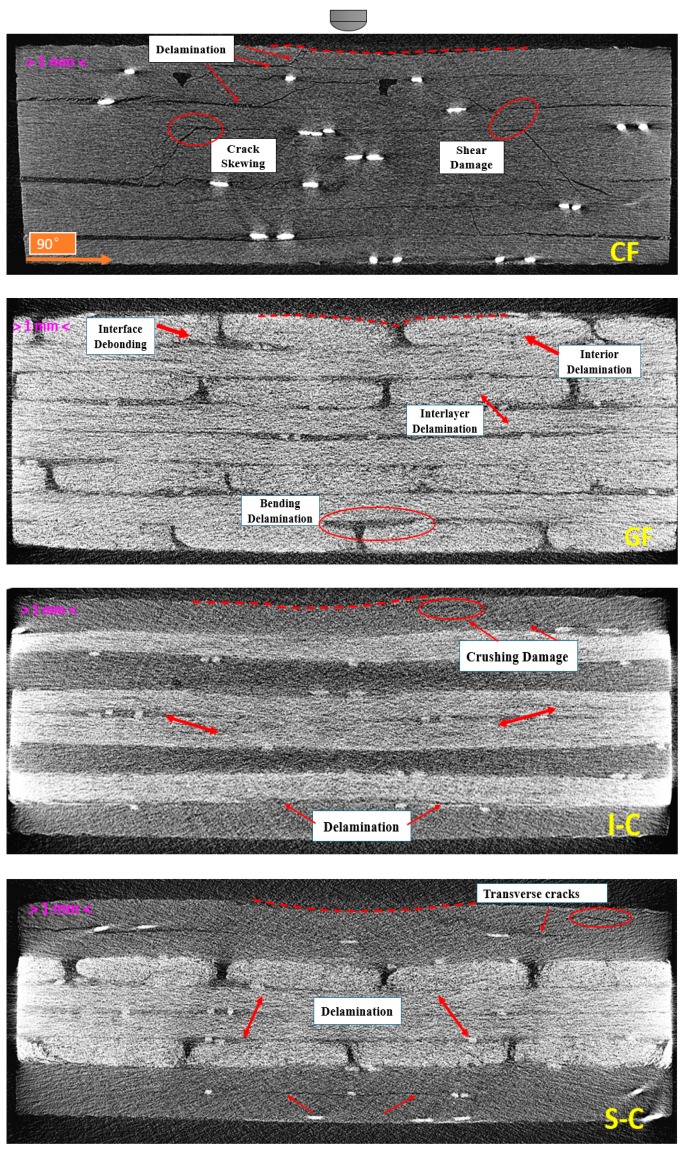
Representative CT scans (0° slices) of the CF, GF, I-C, and S-C specimens after a 30 J impact.

To get a better insight into the damage modes of the laminates, a CT-scan analysis was performed on CF, GF, I-C (maximum peak load), and S-C (maximum energy absorption) laminates after 30 J impact. As is shown in Figure 7, CF exhibited a typical “pine tree” damage pattern [35]: the crushing fracture and shear cracks caused by compression stress propagated conically through the thickness of the laminate, whereas skewing at the interface resulted in shear stress among two plies, leading to the delamination. The stress waves propagated upward along the gap of fiber bundle due to the bending stress on the back surface of GF, causing delamination. The fibers on the front surface were deformed after the impact, and some severe fiber-matrix debonding was found. I-C and S-C exhibited a similar damage pattern (including fiber breakage in the top ply and delamination in the bottom ply), but not as seriously, indicating the interlayer hybrid structure played a positive role in improving impact resistance. The front surface of I-C showed a dent with a few brittle failures of carbon fiber, and the integrity of the inner glass fiber layers was good. The damage level was slight with little delamination on the bottom C-G interface. However, the number of C-G hybrid interfaces in S-C was less than that of I-C. The internal cracks in the bottom and top plies grew upwards and downwards, respectively, leading to the delamination propagation, especially on the inner glass layers. In addition, the microcracks in S-C were more than that in I-C, including transverse cracks on the top two carbon fiber plies. Therefore, the damage mode confirmed that the interlayer hybrid structure played a positive role in resisting damage propagation.

The intralayer hybrid composites exhibited the different damage modes with both in-plane and interlayer hybrid interface in contrast to the interlayer hybrid composites without the in-plane hybrid interface. As shown in Figure 1, NCFs consist of carbon and glass fibers, and the width of a single fiber bundle is 5 mm. A 6.3 mm diameter circle can be observed on the front surface after 30 J impact; thus, the “fiber type of the impact location” was taken into account. 

**Figure 8:**
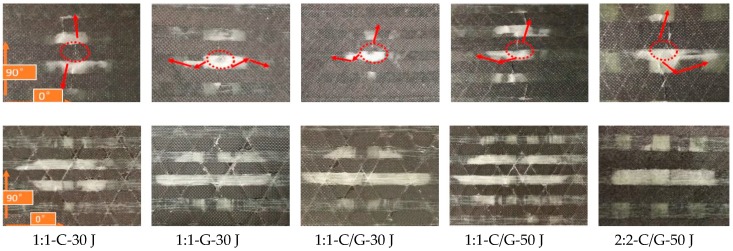
Front and back view photographs of intralayer hybrid composite specimens after impact. Row 1: front, Row 2: back.

The failure modes of the intralayer hybrid composite laminates are shown in Figure 8 (the dashed line represents the impact location and the arrow indicates the stress wave propagation path). As the impact energy and hybrid structure was kept constant, the impact on the carbon fiber resulted in the stress wave going through the glass fiber, inducing the transverse cracks propagated on the adjacent carbon bundles. When the impact location was glass fiber, the stress wave was refracted between the two carbon bundles. The above two types of damage pattern occurred simultaneously while the impact location was the C-G interface, and the damage on the back surface was similar as the impact energy increased, but more broken carbon bundles and glass fiber-matrix debonding could be found on the front surface.

**Figure 9:**
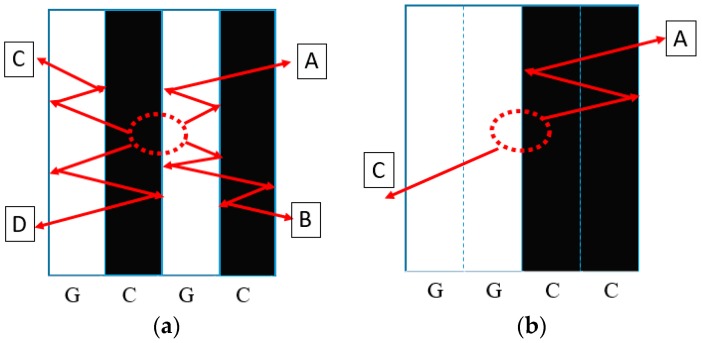
Model of stress wave propagation in intralayer hybrid composite specimens: (**a**) 1:1; (**b**) 2:2.

Figure 9 illustrates the stress wave propagation path when the impact location is the C-G interface (dotted line area) under 50 J impact energy. When the stress wave went through the C-G interface for the 1:1 laminate, four types of transmission and reflection (A–D) occurred due to the small unit width of the fiber bundle. On the other hand, the 2:2 laminate had a larger fiber bundle unit width with a smaller number of C-G hybrid interfaces than the 1:1 laminate per unit area, with only A-type reflection and B-type transmission of stress wave, which showed a lesser attenuation of the stress wave strength, causing more intraply cracking. Thus, the greater number of C-G hybrid interfaces in the 1:1 laminate is related to the higher impact resistance.

### 3.3. Delamination

**Figure 10:**
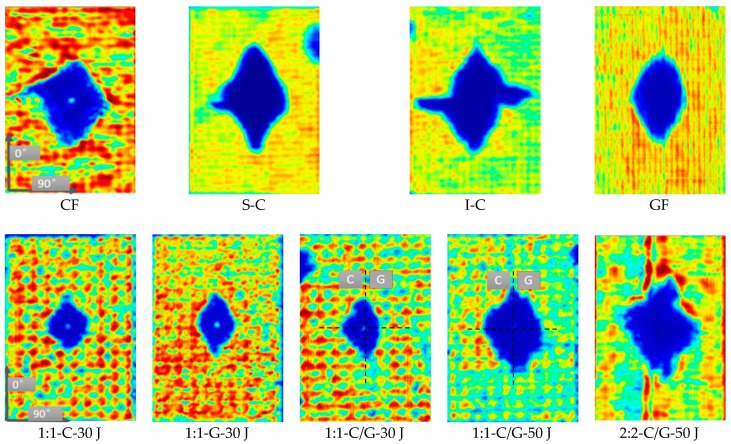
C-scan images of various composite specimens after 50 J impact and intralayer hybrid composite specimens.

**Figure 11:**
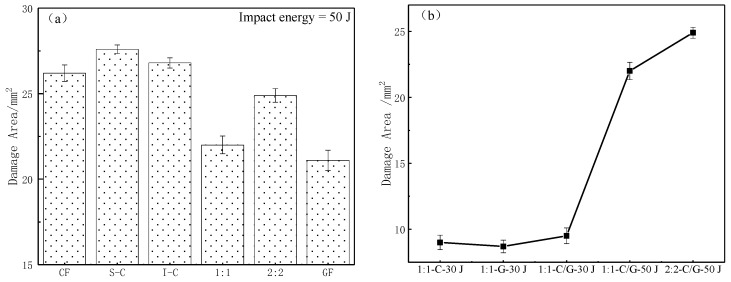
Damage area of (**a**) various composite specimens after 50 J impact and (**b**) intralayer composite specimens.

The C-scan results of the impacted specimens are shown in Figure 10. Due to the bending force, most of the delamination occurred on the bottom ply of the laminate. The stress wave spread fast in the carbon fiber due to the high modulus [36]. Thus, the delamination exhibited the diamond shape since the carbon fiber was used as the outer surface. The delamination area obtained via C-scan is summarized in Figure 11a. The S-C had the largest damage area with the highest energy absorption.

The internal damage mode of the intralayer hybrid composites was affected by the impact location and hybrid structure. The delaminated area was measured in Figure 11b. A diamond shape delamination was observed when the impact location was carbon fiber. On the other hand, glass fiber showed a long narrow delamination with minimal area as the impact point. On the other side impacting at the C-G interface, the damage image revealed a “peak” shape on the carbon fiber side, whereas the glass fiber side was flatter, showing an asymmetric mode with the largest delamination area. As expected, the delamination area increased with increasing impact energy but the asymmetry decreased. The delamination area of the 2:2 laminate was larger than that of the 1:1 laminate, which could explain the higher energy absorption of the 2:2 laminate. It should be noted that the delamination damage affected by the hybrid structure is greater than that of the impact location, as compared in Figure 11b.

Specifically, the peak load of 1:1 was similar to I-C, whereas it exhibited a smaller damage area under the same impact energy, which was due to the in-plane and interlayer C-G hybrid interface in the laminate. Therefore, the 1:1 hybrid structure revealed a better impact resistance performance as compared with the other hybrid structures.

## 4. Conclusions

The behavior of interlayer/intralayer hybrid composites based on carbon and glass non-crimp fabric under low-velocity impact was studied. 

The interlayer hybrid structure exhibited a better impact performance compared to the sandwich hybrid structure due to the more numerous of C-G hybrid interfaces. I-C showed the biggest peak load with small damage, whereas the S-C showed the highest energy absorption with more internal damage. A better impact resistance can be achieved by deploying a suitable C-G interface.

The intralayer hybrid structure had in-plane and interlayer C-G hybrid interfaces, resisting the propagation of the stress wave in both transverse and longitudinal directions. The peak load of the 1:1 was slightly less than that of the CF due to the small unit width of the fiber bundle. In contrast, the 2:2 structure decreased the resistance to the stress wave propagation and improved the energy absorption.

The delamination of the interlayer hybrid structure was affected by the stacking sequence. The S-C structure had the largest delamination area. In addition, the internal damage of the intralayer hybrid structure was affected by the impact location and the hybrid structure, the hybrid structure having a dominant effect. 

The 1:1 structure had a higher peak load and the smaller damage area compared to I-C under the same hybrid ratio and impact energy level, indicating a higher impact resistance as compared to the other hybrid structures. In general, a better impact resistance can be achieved by designing an appropriate intralayer hybrid structure with a constant hybrid ratio. Meanwhile, because hybrid composites will be used over the long term, later studies should investigate the stability property.

## Figures and Tables

**Table 1:** Hybrid fabric specifications.

Fabric Type	Areal Density (g/m^2^)		Ratio of C/G
	Carbon Fiber	Glass Fiber	
Carbon	728.3	0	1:0
Glass	0	944.9	0:1
C-G	364.2	472.4	1:1
C-C-G-G	364.2	472.4	1:1

**Table 2:** Stacking configurations of hybrid structures.

Structure	Non-Hybrid		Interlayer
Stacking sequences	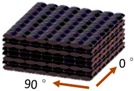	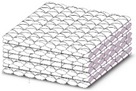	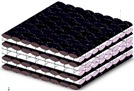
Nomenclature	CF	GF	I-C
**Structure**	**Sandwich**	**Intralayer**	
Stacking sequences	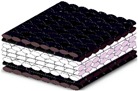	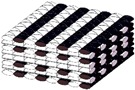	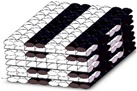
Nomenclature	S-C	1:1	2:2

Note: CF and GF denote the pure carbon and glass fabric layer, respectively. I-C represents the interlayer hybrid structure with the carbon fiber on the impact surface. S-C represents the sandwich hybrid structure with the carbon fiber on the impact surface. 1:1 and 2:2 represent the intralayer hybrid structure formed by the C-G fabric.
